# Recent Progress Regarding Jasmonates in Tea Plants: Biosynthesis, Signaling, and Function in Stress Responses

**DOI:** 10.3390/ijms25021079

**Published:** 2024-01-16

**Authors:** Xin Zhang, Yongchen Yu, Jin Zhang, Xiaona Qian, Xiwang Li, Xiaoling Sun

**Affiliations:** 1 Tea Research Institute, Chinese Academy of Agricultural Sciences, No. 9 South Meiling Road, Hangzhou 310008, China; xinzhang@tricaas.com (X.Z.); yuyongchen@tricaas.com (Y.Y.); zhangjin1369@tricaas.com (J.Z.); qianxiaona@tricaas.com (X.Q.); lixiwang0392@tricaas.com (X.L.); 2 Key Laboratory of Biology, Genetics and Breeding of Special Economic Animals and Plants, Ministry of Agriculture and Rural Affairs, Hangzhou 310008, China

**Keywords:** jasmonates, biosynthesis, tea plant, defense response, biotic stress, abiotic stress

## Abstract

Tea plants have to adapt to frequently challenging environments due to their sessile lifestyle and perennial evergreen nature. Jasmonates regulate not only tea plants’ responses to biotic stresses, including herbivore attack and pathogen infection, but also tolerance to abiotic stresses, such as extreme weather conditions and osmotic stress. In this review, we summarize recent progress about jasmonaic acid (JA) biosynthesis and signaling pathways, as well as the underlying mechanisms mediated by jasmontes in tea plants in responses to biotic stresses and abiotic stresses. This review provides a reference for future research on the JA signaling pathway in terms of its regulation against various stresses of tea plants. Due to the lack of a genetic transformation system, the JA pathway of tea plants is still in the preliminary stages. It is necessary to perform further efforts to identify new components involved in the JA regulatory pathway through the combination of genetic and biochemical methods.

## 1. Introduction

Plants are consistently exposed to various biotic and abiotic stresses in the natural environment due to their sessile lifestyle. To ensure survival under diverse and challenging conditions, plants have developed an intricate and adaptable network to respond to environmental stimuli [1,2]. Hormones play a crucial role in improving the ability of plants to adapt to these stresses, regulating nearly all aspects of development, physiology, and metabolism. Among these, fatty-acid-derived jasmonates, including jasmonic acid (JA) and its various derivatives, have attracted much attention for mediating plant responses to both abiotic and biotic stress [3,4,5]. Over the past two decades, there has been significant progress in understanding the molecular mechanisms underlying JA biosynthesis and signaling, especially in model plants such as Arabidopsis (*Arabidopsis thaliana*) and tomato (*Solanum lycopersicum*) [1,6,7,8]. In addition, JA interacts with different hormone signaling pathways to mediate diverse plant defense responses and various developmental processes in plants, such as salicylic acid (SA), gibberellic acid (GA), ethylene (ET), and abscisic acid (ABA) [1,4,6].

The tea plant *Camellia sinensis* (L.) O. Kuntze is one of the world’s most important woody cash crops that can be used to produce non-alcoholic beverages [9]. It is highly valued for its secondary metabolites, such as catechins, theanine, and diverse volatile compounds, which not only contribute to form the unique characteristics of tea, such as color, flavor, and taste, but also help to adapt to various environmental stresses [10,11]. Under natural conditions, tea plants are susceptible to a wide range of biotic stresses caused by herbivorous insects and microbial pathogens, including tea geometrid (*Ectropis grisescens*, *E. obliqua*), tea green leafhopper (*Empoasca* (*Matsumurasca*) *onukii* Matsuda), *Thrips hawaiiensis* (Morgan), and the pathogenic fungus anthracnose (*Colletotrichum*), alongside abiotic stressors caused by adverse climatic conditions. Those biotic and abiotic stressors often cause a considerable decrease in tea yield and quality worldwide [9,11,12]. A growing body of research suggests that the activation of the JA signaling pathway is linked to defense responses of the tea plants to different types of stresses [13,14,15]. These observations have led to the demand for elucidating the action mechanisms of jasmonate in tea plants and have resulted in a series of research findings in the field of JA signaling pathways. Here, this review outlines the current knowledge on JA biosynthesis and signaling components, as well as its function and mechanism in mediating tea plants’ defenses against biotic and abiotic stresses.

## 2. Research Advances in JA Biosynthesis

**Figure 1:**
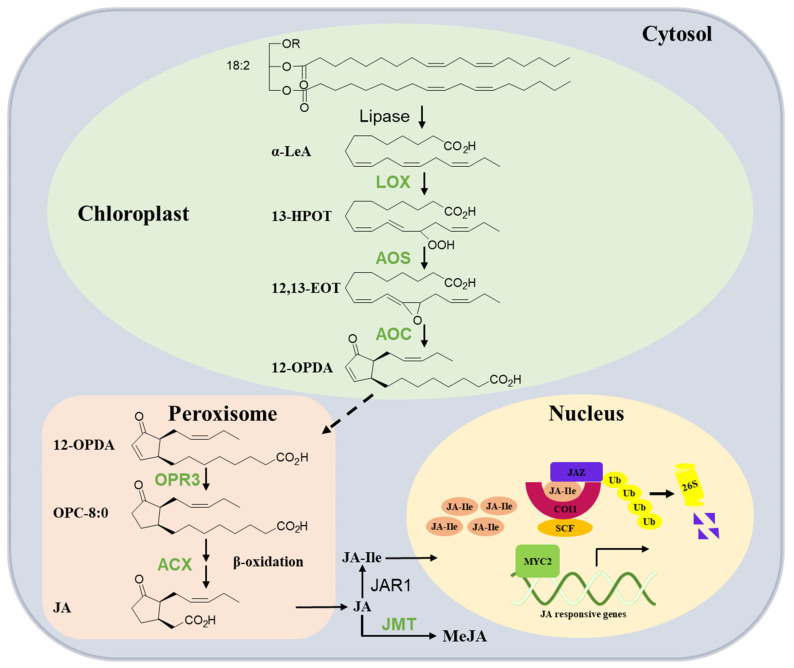
Overview of JA biosynthesis and signaling pathway in tea plants. In chloroplasts, polyunsaturated fatty acids are converted to 12-oxophytodienoic acid (12-OPDA) by the sequential activities of several enzymes including lipoxygenase (LOX), allene oxide synthase (AOS), and allene oxide cyclase (AOC). 12-OPDA is then transported to the peroxisome and reduced by OPDA reductase3 (OPR3) to 3-oxo-2-(2′(Z)-pentenyl)-cyclopentane-1-octanoic acid (OPC-8:0); OPC-8:0 undergoes β-oxidation by acyl-CoA oxidase (ACX) to yield JA; in the cytosol, JA is converted to JA-Ile by jasmonoyl-isoleucine synthetase (JAR1) and MeJA by JA methyltransferase (JMT). JA-Ile is transported to the nucleus and perceived by the COI1-JAZ co-receptor complex upon stimulation (high JA-Ile levels). Jasmonate ZIM domain proteins (JAZs) are recruited by COI1 and subjected to ubiquitinylation and subsequent degradation by the 26S proteasome. Subsequently, bHLH transcription factor (MYC2) can activate the transcription of early JA-responsive genes such as those encoding JAZ and MYC2. SCF complex: complex consisting of Skp1, Cullin-1, and F-box protein; Ub: ubiquitin. See the explanations in the text. The enzymes reported to be involved in JA biosynthesis in the tea plant (blue) are shown.

The synthesis of JA was initially elucidated by Vick and Zimmermann [16]. Subsequent investigations and reviews have extensively summarized the pathway for JA biosynthesis in plants. Therefore, this section offers a brief summary of our current knowledge (Figure 1). JA production is completed by a multi-step enzymatic process that occurs sequentially in the chloroplast, peroxisome, and cytoplasm. The fatty acid substrate of JA originates from the release of α-linolenic acid 18:3 (α-LeA) from galactolipids in chloroplast membranes. Then, the pathway is initiated by the lipoxygenase (LOX)-catalyzed oxygenation of α-LeA. The LOX product 13(*S*)-hydroperoxy-octadecatrienoic acid (13-HOPT) is converted by allene oxide synthase (AOS), leading to the generation of an unstable allene oxide, 12,13(S)-epoxy-octadecatrienoic acid (12,13-EOT). The subsequent enzyme, allene oxide cyclase (AOC), cyclizes 12,13-EOT to produce the 9S, 13S isomer of 12-oxo-phytodienoic acid (12-OPDA), which is the final product of the chloroplast-located part of JA biosynthesis. The next steps take place in the peroxisome, where OPDA is activated and reduced to 10,11-dihydro-12-oxo-phytodienoic acid (OPC-8:0) by 12-oxo-phytodienoatereductase isoenzyme 3 (OPR3). This reaction is followed by fatty acid b-oxidation enzyme acyl-CoA oxidase (ACX1), yielding (+)-7-*iso*-JA, which rearranges into the (−)-JA isomer (with molar ratio of 9:1 for (−)-JA/(+)-7-*iso*-JA). In the cytosol, free JA can be further metabolized into a series of metabolites. For example, it can be conjugated with isoleucine to form JA-Ile by jasmonate-amido synthetase (JAR1) and methylated to the methyl ester of JA (MeJA) by JA carboxyl methyltransferase (JMT). JA-Ile is the bioactive form of JA that is recognized by the JA receptor complex [6,16,17,18,19].

In general, the specific members of the enzymes involved in JA biosynthesis in the tea plant are less clearly defined compared to Arabidopsis and tomato. Most of the genes related to JA biosynthesis in tea plants have been functionally characterized in vitro, as a genetic transformation system for tea plants has not yet been established (Table 1). CsLOX3 contains all the typical plant LOX domains and an N-terminal chloroplast transit peptide. A phylogenetic analysis showed that CsLOX3 shared high similarities with type II LOXs proteins, which are commonly located in chloroplasts. In addition, its heterologous expression protein converted linolenic acid into 13-HPOT by SP-HPLC analysis and then could be accordingly named as 13S-LOX. *CsLOX3* was also induced by mechanical damage, MeJA, SA, and tea geometrid treatments [20]. With the continuous release of the tea genome sequence, more and more genes in the tea plant were identified on a genome-wide scale. Eleven *CsLOX* genes were detected based on the tea tree draft level genome database, and their enzymatic activities and expression characteristics were studied. Among them, expressions of 13-LOX type II subfamily members *CsLOX6* and *CsLOX7* were upregulated after attack by the insect *Ectropis obliqua* and 4 °C cold stresses. *CsLOX6* is predominantly expressed in flowers, and its expression was 105-fold higher relative to that of *CsLOX1*, which was reported to predominantly function in flowers. *CsLOX6* may be a predominant gene involved in the production of JA in tea plant flowers. In addition, a strong induction of *CsLOX7* transcripts was observed in response to MeJA. Then, *CsLOX6* and *CsLOX7* were speculated to be involved in the biosynthesis of JA [21]. *CsAOS2* found in tea flowers was responsive to the damage of thrips. A subcellular localization analysis showed that it targets the chloroplast membrane, suggesting that it may function in the chloroplast. Furthermore, with the transient overexpression of *CsAOS2* in tobacco, the increase of JA in *CsAOS2*-overexpressed plants was significantly higher than that in control after mechanical damage. This suggests that *CsAOS2* may be involved in the synthesis of wound-induced JA [22]. Full-length cDNA and the genomic DNA of a *CsAOC* were isolated, and ChloroP 1.1 analysis predicted that its protein contained a chloroplast signal peptide. *CsAOC* also responded to MeJA, SA, tea leafhopper, and tea geometrid feeding treatment. These results suggested that *CsAOC* may be involved in JA biosynthesis, but further study is needed to confirm [23]. CsOPR3 contained a peroxisomal signal peptide (SRL) at the C-terminus of the protein, a feature shared with other OPR3. The CsOPR3-His recombinant protein reduced *cis*-OPDA and showed a strong preference for (+)-*cis*-OPDA. Transcript expression and protein levels of CsOPR3 were induced by JA, mechanical damage, and tea leaf hopper treatment. In addition, the overexpression of *CsOPR3* in Arabidopsis *opr3* complemented JA synthesis induced by mechanical damage. These observations confirmed the function of *CsOPR3* in JA biosynthesis and its defense role in tea plants’ defense against biotic stress [24]. *CsACX1* and *CsACX3,* isolated from tea plants, can be induced by mechanical wounding, the application of JA, and infestation by tea geometrid and tea green leafhopper. In addition, infection with *Colletotrichum gloeosporioides* (Cgl) could induce the expression of *CsACX3*, but not *CsACX1*. Heterologously expressed protein CsACX1 showed a preference for C_12_ to C_16_-CoA substrates, whereas CsACX3 showed a preference for C_8_ to C_14_-CoA substrates. The overexpression of *CsACX1* or *CsACX3* rescued wound-induced JA synthesis in the Arabidopsis mutant *acx1*. However, only *CsACX3-OE* could enhance the JA synthesis induced by Cgl. *CsACX1* and *CsACX3* showed overlapping functions and distinct roles in the wound- and pathogen-induced JA synthesis, supporting the view that different enzyme isoforms have distinct physiological functions [25,26]. 

MeJA is a crucial volatile organic compound in tea plants as it imparts a high-quality floral fragrance to tea beverages. *CsJMT* was cloned from tea leaves and its recombinant protein catalyzed the substrates of JA and SAM into volatile product MeJA. In addition, site-directed deletions revealed that N-10, S-22, and Q-25 residues in the beginningamino acids played a key functional role in CsJMT enzyme activity. Subcellular localization analysis indicated that the CsJMT protein is located in the cytoplasm [27]. The accumulation of 1R, 2R-MeJA was found to correlate with *CsJMT* expression during the shaking process of oolong tea. Further investigation showed that mechanical injury and low-temperature stress could significantly induce *CsJMT* expression and MeJA accumulation after harvest [28]. 

**Table 1:** Comparison of major components of JA pathway between tea plants and other species.

Gene	Gene ID of Tea Plants	Gene Number in Tea Plants	Functions in Tea Plants	Functions in Other Species	References
*LOX*	HM440161 (Genbank)	1	located in chloroplast; converted LeA into 13-HPOT; induced by wounding, MeJA, SA, and tea geometrid treatments [20]	wound-induced JA biosynthesis [29]; defense against herbivores [30]; resistance to *Botrytis cinerea* [31]	[20,21,22,23,24,25,26,27,28,29]
*AOS*	114307969	1	target the chloroplast, wound-induced JA biosynthesis with *CsAOS2* transit overexpression [22]	wound-induced JA biosynthesis and male sterility [32]; resistance to *Erwinia carotovora* [33]	[22,32,33]
*AOC*	114307969	1	respond to MeJA, SA, tea leaf hopper, and tea geometrid treatment [23]	resistance to the blast fungus [34]; wound-induced JA biosynthesis [35]	[23,34,35]
*OPR3*	114299110	1	(+)-*cis*-OPDA reductase activity [24]; restore wound-induced JA biosynthesis in Arabidopsis *opr3* plants [14]	male sterility [36]; JA biosynthesis [37]	[14,24,26,27,28,29,30,31,32,33,34,35,36,37]
*ACX*	114279706; 114266146	2	induced by wounding, JA, tea geometrid and the tea green leafhopper treatments; restore wound-induced JA biosynthesis in *acx1* plants [26]	wound-induced JA biosynthesis; defense against tobacco hornworm [38]	[26,38]
*JMT*	114288630	1	located in cytoplasm [27]; catalyze JA and SAM into MeJA [28]	resistance against fungus *Botrytis cinerea*; seed germination and mass [39]	[27,28,39]
*JAZ1*	114317541	1	involved in synthesis of flavonoid [40,41]	interact with different proteins to modulate JA regulated physiological process [42,43,44,45,46]	[40,41,42,43,44,45,46]
*MYC2*	114266177	2	involved in accumulating multiple JA-regulated defense compounds [23,40,47,48,49,50,51,52]	modulates diverse JA-dependent functions: development, and pathogen/wound response, etc. [6,18,19,53,54]	[6,18,19,23,40,47,48,49,50,51,52,53,54]

## 3. Research Advances in JA Signaling Transduction

Decades of studies on the model system of Arabidopsis have uncovered the core components in JA signaling, including the F-box protein COI1 that links JA signaling to Skp-Cullin-F-box (SCF) E3 ubiquitin ligase complex, the Jasmonate-ZIM domain (JAZ)-repressor proteins, and a variety of transcription factors that participate in diverse JA responses [18,19,55]. At resting stage, JAZ proteins interact with key transcription factors (i.e., MYCs and EINs) and inhibit their transcriptional activity. Upon environmental stresses or developmental signals, JA-Ile is rapidly synthesized and recognized by the COI1-JAZ receptor complex, leading to the degradation of JAZs via the 26S proteasome and release transcription factors to modulate various JA-responsive genes (Figure 1).

The discovery of JAZ proteins in 2007 has led to remarkable progress in understanding how the biosynthesis of JA-Ile is linked to the transcriptional activation of JA responsive genes [42,43,44,55]. Based on different levels of tea genome, three research groups have described and studied the gene members encoding JAZ proteins in tea plants. In addition to basic information, such as the protein structure, subcellular localization, and response pattern to JA, they also explored the responses patterns of *CsJAZs* to abiotic stresses, biotic stresses, and postharvest processing treatment, and the above preliminary studies also suggested the complexity and diversity of the functions of the JAZ proteins in tea plants [45,56,57]. Subsequently, different isoforms of CsJAZ1 were found to be negatively associated with JA-mediated flavan-3-ol biosynthesis in the tea plant. *CsJAZ1* is selectively spliced post-transcriptionally, and the same transcript can encode protein with full length (CsJAZ1-1), protein lacking 3′ coding sequences (CsJAZ1-2), and protein with the complete Jas domain missing (CsJAZ1-3), resulting in JAZs with different functions. CsJAZ1-2 acts as an alternative enhancer to CsJAZ1-1 and an antagonist to CsJAZ1-1 when binding to CsMYC2.1. In the presence of JA, CsJAZ1-3 interacted with CsJAZ1-1 and CsJAZ1-2 to form heterodimers. These heterodimers stabilized the CsJAZ1-1-CsMYC2.1 and CsJAZ1-2-CsMYC2.1 complexes, thereby repressing the transcription of four genes that act late in the flavan-3-ol biosynthetic pathway. The suppression of three *CsJAZ1* expressions increases the content of epicatechin-3-gallate (ECG) and epigallocatechin-3-gallate (EGCG) in tea leaves. The overexpression of *CsJAZ1-3* with no Jas domain in Arabidopsis showed that the content of anthocyanins in leaves significantly increased after JA treatment [40].

The basic helix–loop–helix (bHLH) protein MYC2 and its close relatives, such as MYC3 and MYC4, are the most intensively studied JA-inducible transcription factors (TFs). They bind to G-box motifs to regulate the expression of a large portion of JA-responsive genes [46,53,54]. To date, the MYC2-like proteins functionally reported in tea plants are CsMYC2.1 (TEA000833.1), CsMYC2 (XM_028207058.1), CsMYC2a (MK336383), and CsMYC2.2 (TEA003964.1) [40,41,47,58]. Sequence comparison suggests that CsMYC2.1, CsMYC2, and CsMYC2a encode the same protein. To avoid confusion with CsMYC2.2, the subsequent text will uniformly refer to CsMYC2.1, CsMYC2, and CsMYC2a as CsMYC2.1. Overexpressed *CsMYC2.1* partially compensated for JA sensitivity in the Arabidopsis *myc2* mutant, which was evidenced by the root inhibition phenotype, the expression of MYC2-regulated genes, and anthocyanins accumulation in the presence of JA. The results suggest the function of CsMYC2.1 in JA-mediated flavonoid biosynthesis and root growth responses [40,58]. Recent studies have shown that CsMYC2 functions as the foremost regulatory hub in the JA signaling pathway, facilitating multiple JA-mediated physiological processes. More details will be described in a later section of the article.

## 4. Defense Responses Regulated by JA in Tea Plants

### 4.1. JA Regulation in Biotic Stress Process

Infestations of tea geometrid or tea green leafhopper caused a significant increase in the accumulation of JA and JA-Ile, as well as the expression of JA-associated genes in tea plants [9,12,59]. Caterpillars of *E. obliqua* or *E. grisescens*, which were fed on tea plants treated with JA or MeJA, exhibited slower growth and development than those fed on control plants [9,59]. Additionally, the application of MeJA in tea plants significantly enhanced the attractiveness to female adults of *Apanteles* sp. It also increased the parasitized rate of *E. obliqua* larvae in the field [13].

**Figure 2:**
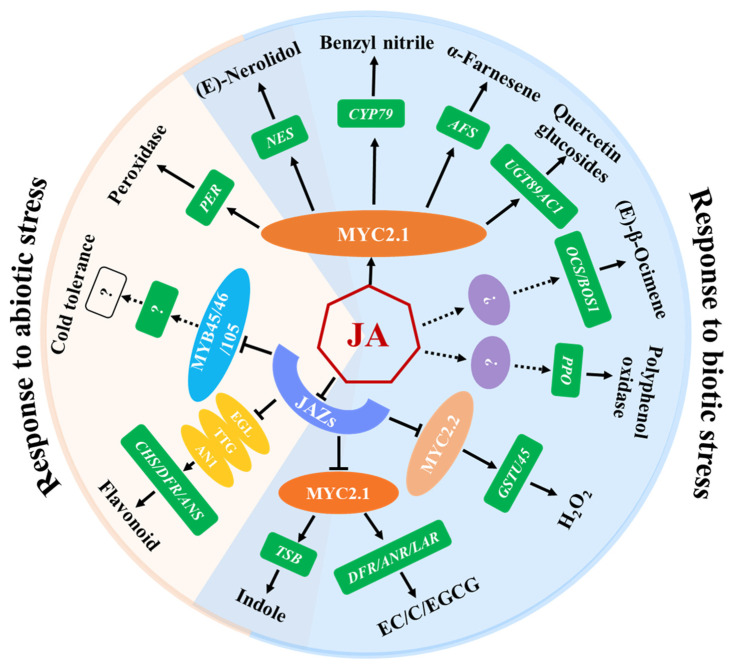
Functions of JA: regulating defense compounds of tea plants that are involved in biotic and abiotic stresses. JAZ proteins act as repressors of JA signaling by binding and repressing a series of transcription factors, MYC2.1/2.2, EGL/TTG, and MYB45/46/105, which are essential for the corresponding JA responses. MYC2.1/2.2 and EGL/TTG regulate the expression of target genes and the accumulation of metabolites in response to various stresses. Metabolites involved in abiotic stresses are included in the region highlighted in light pink, while the region highlighted in light blue indicates metabolites involved in biotic stresses. The overlapping regions represent metabolites involved in both biotic and abiotic stresses.

Although numerous inducible secondary metabolites have been identified through untargeted metabolomics of tea plants, only a few compounds have been clarified for their functions in tea plant defenses against pests. JA modulates the defense mechanisms that stimulate the biosynthesis of non-volatile defense compounds, which exert direct toxic and anti-nutritional effects on pests of tea plants (Figure 2, Table 2) [60,61,62,63,64]. Polyphenol oxidases (PPOs), known as inducible defense proteins, contribute to tea plant resistance against tea geometrid larvae and their expression and activity is positively regulated by JA [15]. In addition, epicatechin, (+)-catechin, and EGCG induced by *E. grisescens* feeding are important inducible defensive compounds against tea geometrid larvae in tea plants, whose accumulation is partly dependent on the JA signaling pathway [9]. Quercetin glucosides, the glucosylated form of quercetin catalyzed by UGT89AC1, is another defensive compound identified to reduce the larval growth of *E. grisescens*. JA, JA-Ile, and MeJA upregulate the expression of *CsUGT89AC1* and promote the production of quercetin glucosides. In addition, a MYC-binding cis-element is detected in the Cs*UGT89AC1* promoter analyzed by the online PLACE tool, and the expression of *CsUGT89AC1* significantly decreased in *CsMYC2.1*-silenced tea plants compared to control plants, suggesting that *CsUGT89AC1* is a target gene of CsMYC2.1. Therefore, the JA signaling pathway may induce the accumulation of quercetin glucosides through CsMYC2.1, thereby enhancing the resistance of tea plants to *E. grisescens* [65].

Herbivore-induced plant volatiles (HIPVs) play a vital role in regulating the tritrophic interactions of herbivores, natural enemies, and plants [66]. To date, multiple kinds of tea plant HIPVs, such as green leaf volatiles, terpenoids, indole, and nitrogen-containing compounds, have been demonstrated to be induced by the infestation of the tea geometrid, tea green leafhopper, or tea weevil, to directly deter herbivores or attract the herbivore’s natural enemies [67,68]. For example, monoterpenes (linalool and β-ocimene), two sesquiterpenes (α-farnesene and (*Z*)-nerolidol), a green leaf volatile ((*Z*)-3-hexenol), a nitrogen-containing compound (benzyl nitrile), and indole were emitted at higher concentrations when the tea plants were attacked by the tea tortrix (*Adoxophyes honmai* Yasuda). The exogenous application of JA to tea leaves induced a volatile mixture that was similar, although not identical, to that induced by the smaller tea tortrix [69]. Those active compounds that exhibit direct behavioral regulation functions on the conspecific pests and their natural enemies have been comprehensively studied. Furthermore, the regulatory mechanisms of JA on the synthesis of benzyl nitrile, indole, α-farnesene, nerolidol, and β-ocimene were elucidated (Figure 2, Table 2). Benzyl nitrile, a nitrile-containing phenylpropanoid/benzenoid volatile, exhibits a notable increase in tea plants that are infested by tea geometrids. The Y-tube olfactometer assay and insect resistance analysis revealed that benzyl nitrile can repel tea geometrids’ larvae and inhibit their growth. *CsCYP79* was shown to regulate the biosynthesis of benzyl nitrile in transiently transformed *Nicotiana benthamiana* plants. The JA-related transcription factor CsMYC2.1 serves as an activator of *CsCYP79* under damage conditions. The study revealed that herbivore-induced damage depends on the JA signaling pathway for the synthesis and release of benzyl nitrile, which protects plants from diurnal herbivorous tea geometrid larvae [48].

Furthermore, several HIPVs released from pest-infested tea plants can serve as volatile signals to convey information or elicit defense responses in undamaged tissues or neighboring plants. Volatile indole acts as a priming signal for tea plants to prepare themselves for the attack of the forthcoming tea geometrid caterpillars. Interestingly, Ca^2+^ and jasmonate signaling have been verified to be required for indole-mediated defense priming and herbivore resistance [49]. The exogenous application of JA promoted the production of both internal and emission indole. CsTSB2, which encodes a tryptophan synthase β-subunit, is essential for indole synthesis and its gene expression in tea leaves and was upregulated by JA, which is consistent with the accumulation of indole. CsMYC2.1 regulates indole biosynthesis in JA signaling by positively regulating the expression of *CsTSB2*, while CsJAZ2 significantly inhibits the induction of *CsTSB2* by CsMYC2.1 [47]. (*E*)-Nerolidol not only repels mated *E. obliqua* females, but also acts as a signal to induce tea plant defenses against pathogens and insects and to modulate cold stress tolerance in tea plants [14,70]. Treatment with JA significantly increased the *CsNES* expression and (*E*)-nerolidol emission in tea leaves, showing a similar effect to that of the tea green leafhopper infestation. The JA core transcription factor CsMYC2.1 directly targets the *CsNES* promoter. Additionally, histone deacetylase 2 (CsHDA2) interacted with CsMYC2.1 to co-regulate *CsNES* expression and (*E*)-nerolidol production [71]. β-ocimene strongly repels mated *E. obliqua* females and indirectly interferes with tea geometrid growth via signaling [70,72]. In addition, β-ocimene alters the metabolite profiles of the neighboring undamaged tea leaves [50]. In tea plants, combined treatments with mechanical damage and JA application induce the de novo synthesis of (*E*)-β-ocimene. Treatment with exogenous MeJA also increases the transcript level of *CsOCS*, suggesting that the elevated level of JA resulting from multiple stresses enhances the expression of *CsOCS*, leading to the accumulation of β-ocimene to high levels [73]. The accumulation of α-farnesene is significantly induced by the infestation of tea geometrid, tea green leafhopper, or tea tortrix. α-Farnesene attracts the natural enemies, wasps (*Vespabicolor Fabricius*), of the tea geometrid and helps tea plants in their defense against infestation via increasing the emission of β-ocimene from neighboring tea plants, to repel moth preference. It has been further proven that the induction of β-ocimene by α-farnesene is dependent on Ca^2+^ and JA signaling [70]. JA acts as a key upstream hormone regulating the α-farnesene synthesis gene *CsAFS* through the CsHDA6-CsMYC2.1 transcriptional regulatory module upon herbivore infestation. CsMYC2.1 regulates α-farnesene synthesis by activating the expression of *CsAFS*. Additionally, histone deacetylases CsHDA6 can directly interact with CsMYC2.1 to co-regulate α-farnesene synthesis in tea plants. CsHDA6 also exhibits HDAC activity and affects the acetylation levels of modifications on JA-induced *CsAFS* expression [51]. In summary, the accumulated studies further demonstrate that JA is a crucial signal transduction pathway for modulating the emission of HIPVs from tea plants.

**Table 2:** JA-regulated defensive compounds in tea plants.

Compounds	Functions	Regulatory Mechanism by JA	References
Polyphenol oxidases	reduce the larvae growth of *E. grisescens*	upregulate the *CsPPO* expression and increase the CsPPO activity	[15]
Catechins (EC, C, EGCG)	reduce the larvae growth of *E. grisescens*	increase the content of EC, C, and EGCG [9]; regulate expression of *CsDFR/CsANR/CsLAR* with CsJAZ1-CsMYC2.1 signaling module [40]	[9,40]
Quercetin glucosides	reduce the larvae growth of *E. grisescens*	induce the expression of biosynthesis gene *CsUGT89AC1* via CsMYC2.1	[65]
Indole	pre-exposure increases tea resistance to *E. obliqua* larvae	promote the production of indole, regulate *CsTSB2* expression with CsJAZ2-CsMYC2.1 signaling module	[47]
α-Farnesene	attracting natural enemy wasps of *E. grisescens* larvae; alter the metabolite profile in the neighboring tea leaves; induce the emission of β-Ocimene in neighboring tea plants	regulate *CsAFS* expression through CsHDA6-CsMYC2.1 signaling module	[51]
Benzyl nitrile	repel *E. grisescens* larvae and inhibit their growth	*CsCYP79* is the target gene of CsMYC2.1 and is positively regulated by CsMYC2.1	[23]
(*E*)-Nerolidol	induce plant defenses against pathogens and insects [14]; repel mated *E. obliqua* females [70]; participate in cold stress tolerance [71]	increase the *CsNES* expression and (*E*)-nerolidol content through CsMYC2.1 [71]	[14,70,71]
β-Ocimene	alter the metabolite profile of neighboring tea plants [50]; repel mated *E. obliqua* females [70]; induce plant resistance against *E. grisescens* larvae [71];	increase the expression of *CsOCS* and *CsBOS1* and β-ocimene content [70,73]	[50,70,71,73]
Peroxidase	improve plant tolerance against mannitol stress	regulate expression of *CsPER1* and *CsPER3* via CsMYC2.1	[74]

Fascinatingly, JA is found to be involved in tea plant–pathogens interactions, as well. Integrated transcriptomic and metabolomic analyses demonstrate a significant increase in the levels of JA, 13(*S*)-HPOT, and 12-OPDA, as well as the upregulation of JA biosynthesis-related genes *CsAOS* and *CsAOC* during the late infection of *Colletotrichum camelliae* [75]. Furthermore, infection by *C. fructicola* leads to increased JA and JA-Ile content, and promotes the expression and protein levels of the JA biosynthesis gene *CsOPR3*. Pre-treating tea plants with JA can alleviate the severe cell death of tea plants caused by the infestation of *C. fructicola* [14]. The underlying mechanism by which JA modulates tea plant defenses against *C. camelliae* through regulating *CsGSTU45* has recently been elucidated (Figure 2). CsGSTU45 negatively regulates disease resistance against *C. camelliae* by inducing H_2_O_2_ accumulation. CsMYC2.2 directly binds to and activates the promoter of *CsGSTU45*. Furthermore, *CsMYC2.2*-silenced tea plants show enhanced disease resistance with reduced transcript and protein levels of CsGSTU45, and decreased H_2_O_2_ content. CsJAZ1 interacts with CsMYC2.2 and represses its regulation of the accumulation of CsGSTU45 protein, resulting in the enhanced disease resistance of tea plants. Therefore, the JA signaling pathway modulates tea plant susceptibility to *C. camelliae* by regulating *CsGSTU45* to promote H_2_O_2_ accumulation with the CsJAZ1-CsMYC2.2 signaling module [41].

### 4.2. JA Regulation in Abiotic Stress Processes

In addition to mediating the resistance of tea plants to insects and pathogens, JA also plays a crucial role in regulating the response to abiotic stressors, such as low temperature, high temperature, and osmotic stress (Figure 2).

Due to the frequent occurrence of extreme weather conditions, cold stress and high temperature have become two of the most destructive factors in tea plant cultivation. They adversely affect the growth and development of tea plants, leading to negative impacts on the tea industry. Physiological and biochemical assays have shown that the exogenous application of MeJA effectively improves the tolerance of tea plants to cold stress by promoting reactive oxygen species (ROS) scavenging under cold stress conditions. The transcriptional expression levels of *CsMYB45*, *CsMYB46*, and *CsMYB105* were found to be strongly induced when exposed to a combination of MeJA and cold stress treatment. Furthermore, the exogenous application of MeJA amplified the expression of *CsMYB45*, *CsMYB46*, and *CsMYB105* in *E. coli* and improved the growth and survival rates of recombinant cells compared to those with empty vectors under cold stress. Yeast two-hybrid (Y2H) and bimolecular fluorescence complementation (BiFC) experiments confirmed the interaction of CsMYB46 and CsMYB105 with CsJAZ3, CsJAZ10, and CsJAZ11. All results indicate that CsMYB45, CsMYB46, and CsMYB105 are essential components that influence the response to cold stress through the JA signaling pathway [52]. High temperatures significantly decrease the flavonoid content in tea leaves, while JA partially counteracts the suppression of catechin metabolites caused by high temperature. CsHSFA1a and CsHSFA2 are master regulators in the heat stress response, and they directly bind to the *cis*-element of the *CsJAZ6* promoter and activate the transcription of *CsJAZ6*. CsJAZ6 could represses catechin biosynthesis by directly interacting with CsEGL3 and CsTTG1, and the silencing of *CsJAZ6* in tea leaves causes the upregulation of almost all genes involved in flavonoid biosynthesis and a decrease in catechin content. These findings suggest that JA serves as a central regulator mediating the regulatory effect of high temperature on the flavonoid synthesis pathway [76].

Additionally, JA also regulates the osmotic stress response, which severely inhibits the development and yield of tea plants. An integrated transcriptome analysis revealed that the CsMYC2.1 acts as a central hub in the network induced by mannitol. Its expression positively correlates with the expression of JA biosynthetic genes (*LOX* and *AOS*) and peroxidase (*PER*) genes. In addition, the analysis of protein–DNA interactions indicates that CsMYC2.1 functions as a positive regulator by activating the transcription of *CsLOX7*, *CsAOS2*, *CsPER1*, and *CsPER3* through binding to their respective promoters. The suppression of *CsMYC2.1* expression resulted in a decrease in JA content, peroxidase activity, and osmotic stress tolerance of the tea plant. Meanwhile, the overexpression of *CsMYC2.1* in Arabidopsis enhanced JA content, peroxidase activity, and plant tolerance against mannitol stress. Thus, a positive feedback loop involving CsMYC2.1, CsLOX7, and CsAOS2 enhances osmotic stress tolerance in tea plants through finely tuning the JA accumulation and increasing POD activity [74].

## 5. Conclusions and Prospects

The JA signaling pathway is the vital pathway that modulates tea plants’ defenses against both biotic and abiotic stresses. Although research progress on the jasmonate pathway in tea plants lags behind that of model plants significantly, significant progress has been made in the identification and characterization of the enzymes involved in JA biosynthesis, as well as in understanding the mechanism of induced defense to biotic and abiotic stresses regulated by jasmonates. Thanks to the publication of tea genomes and the development of molecular biology techniques, research on the JA signaling pathway has evolved from isolating individual genes to excavating family members. The interactions between CsJAZs and TFs, such as CsMYC2, CsMYBs, and CsEGL/CsTTG, contribute to JA-mediated transcriptional responses in tea plants.

It is important to recognize that some reports on the JA pathway in tea plants are still in their preliminary stages. The identification systems used to investigate gene function in tea plants are mostly carried out in heterologous expression systems like *E. coli*, yeast, or other model plants. It is necessary to undertake further efforts to identify additional components involved in the JA pathway, such as COI1, MED25, NINJA, and JAR1. The combination of genetic (mutant analyses) and biochemical (enzyme characterization) analyses will considerably advance our understanding of direct evidence for genes participating in JA biosynthesis, signaling transduction, and function in tea plants.

## Data Availability

No new data were created or analyzed in this study. Data sharing is not applicable to this article.
